# A research-based strategy for managing housing adaptations: study protocol for a quasi-experimental trial

**DOI:** 10.1186/s12913-014-0602-5

**Published:** 2014-11-29

**Authors:** Lisa Ekstam, Gunilla Carlsson, Carlos Chiatti, Maria H Nilsson, Agneta Malmgren Fänge

**Affiliations:** Department of Health Sciences, Lund University, Box 157, 221 00 Lund, Sweden; Italian National Research Center on Aging (INRCA), Ancona, Italy

**Keywords:** Context, Disability, Health care, Occupational therapy, Health-economic evaluation

## Abstract

**Background:**

The primary aim of this paper is to describe the design of a project evaluating the effects of using a research-based strategy for managing housing adaptations (HAs). The evaluation targets clients’ perspectives in terms of activity, participation, usability, fear of falling, fall incidence, use of mobility devices, and health-related quality of life, and determines the societal effects of HAs in terms of costs. Additional aims of the project are to explore and describe this strategy in relation to experiences and expectations (a) among clients and cohabitants and (b) occupational therapists in ordinary practice.

**Methods/design:**

This study is a quasi-experimental trial applying a multiphase design, combining quantitative and qualitative data. At the experimental sites, the occupational therapists (OTs) apply the intervention, i.e. a standardized research-based strategy for HA case management. At the control site, the occupational therapists are following their regular routine in relation to HA. Three municipalities in south Sweden will be included based on their population, their geographical dispersion, and their similar organizational structures for HA administration. Identical data on outcomes is being collected at all the sites at the same four time points: before the HA and then 3, 6, and 12 months after the HA. The data-collection methods are semi-structured qualitative interviews, observations, clinical assessments, and certificates related to each client’s HA.

Primary outcomes are the usability of the home and the client’s independence in daily activities (ADL). Cross-sectional and longitudinal data analyses will be conducted as well as statistical analyses, health-economic analyses, and qualitative analyses. Qualitative and quantitative data will be sequentially analyzed, and case studies will be developed.

**Discussion:**

The intervention in this study has been developed and tested through many years of research and in collaboration with practitioners. This process includes methodological development and testing research aimed at identifying the most important outcomes and research targeting current HA case-management procedures in Swedish municipalities. When the study is completed, the results will be used for further optimization of the practice strategy for HA, in close collaboration with the data-collecting OTs.

**Trial registration:**

No: NCT01960582.

## Background

The global prevalence of moderate and severe disabilities is estimated at 15% of the general population and at 50% among those aged 60 and up [1]. To a large extent, disabled persons experience problems in daily life owing to physical and social barriers in the environment [1]; therefore, disability can be reduced by adapting the environment [2].

In Sweden, in compliance with the Housing Adaptation Act [3], housing adaptations (HAs) can be provided to people who experience a declining functional capacity to eliminate physical environmental barriers in the home in order to promote independence and safety. HA in this study are defined according to the Swedish law as adaptation of solid features in the home [3], such as removal of thresholds, installation of grab bars or removal of a bath tub and installation of a shower. HA is a publicly funded intervention, administered by a given municipality in response to a client’s application. The full costs of the HA are granted based on needs assessment and certification by a health professional, most often an occupational therapist (OT) employed by the municipality.

When it comes to outcomes of HA, previous research has indicated that HAs improve independence in terms of activity and participation [4–7], reduce difficulty performing activities [8], and improve the usability of the home [4,5]. In this respect, then, the aims of the intervention seem to have been fulfilled. Moreover, falls and the consequences thereof are common, often resulting in injuries, morbidity, and increased health-care costs [9], but research has shown that fall-related disability is positively affected by HA, particularly among people who have a history of falling [6,10]. Thus, it seems that HAs have the potential to counteract injuries in the home and contribute to increased independence.

Although health deteriorates along an ageing and disablement process, disability may be reduced through the effects of HAs. Higher housing standards and more efficient provision of HAs have been shown to contribute to stability in daily activities and health [11,12]. However, the home also has a symbolic meaning for individuals and their cohabitants, who can be negatively affected by changes to its physical design [13]. Very often, clients who implement HAs also use mobility devices (MDs) indoors, outdoors, or both. MDs are prescribed based on the assumption that they reduce falls and improve activity and participation. Consequently, there is a close link between HA and MD, since prescribing an MD very often calls for an HA, and the two are often negotiated in relation to each other in intervention planning. In spite of this, the combined effects of HA and MD on falls and other disability-related outcomes are to a large extent unknown.

There is a sharp contrast between the relevance of HA and the dearth of economic evaluation of these interventions. Few papers have addressed the costs of assistive technology programs [14–16]. So far only one published study reports a full economic analysis of a home-based intervention that included HA [17]. Although this intervention was cost-effective in terms of life years saved [17], it is difficult to isolate the effect of an HA alone. The interventions compared comprised not only HAs but also a number of other activities. In addition, these findings could have limited generalizability in the Scandinavian context given the differences in costs and funding structures between different countries [18].

When it comes to Sweden, which is the setting for this project, the total cost of the approximately 76,000 HAs approved and funded each year is approximately €115.5 million. Two-thirds of the clients are older people with progressively declining health, whereas younger people and adults with considerable functional limitations comprise a non-negligible group of HA recipients [19]. Thus, the group is quite heterogeneous in terms of functional capacity and health, as well as in terms of housing conditions and standards.

In relation to needs assessment and evaluation, a web-based survey targeting Swedish OTs (N =1,679) [20] showed that most HAs were based on unstructured assessments and non-evidence-based methods and that follow-ups were rarely undertaken. Swedish guidelines for management of the HA process are not based on research and are to large extent insufficient. Consequently, there is a need for research-based, structured strategies for HA case management in Swedish municipalities.

### Hypothesis and aims

The hypothesis of this study is that a new intervention strategy for OTs that takes a research-based and structured approach to assessing and evaluating clients who request new HAs can increase the usability of the home; maintain or increase activity, participation, and health-related quality of life, as well as MD use; and decrease fall incidences and the fear of falling (with respect to the standard approach in ordinary practice). An additional hypothesis is that the new intervention strategy will yield health-economic benefits.

The primary aim of this paper is to describe the design of a project evaluating the effects of using a research-based strategy for managing housing adaptations (HAs). The evaluation targets clients’ perspectives in terms of activity, participation, usability, fear of falling, fall incidence, use of mobility devices, and health-related quality of life, and determines the societal effects of HAs in terms of costs. Additional aims of the project are to explore and describe this strategy in relation to experiences and expectations (a) among clients and cohabitants and (b) occupational therapists in ordinary practice.

## Methods & design

### Design of the study

**Figure 1:**
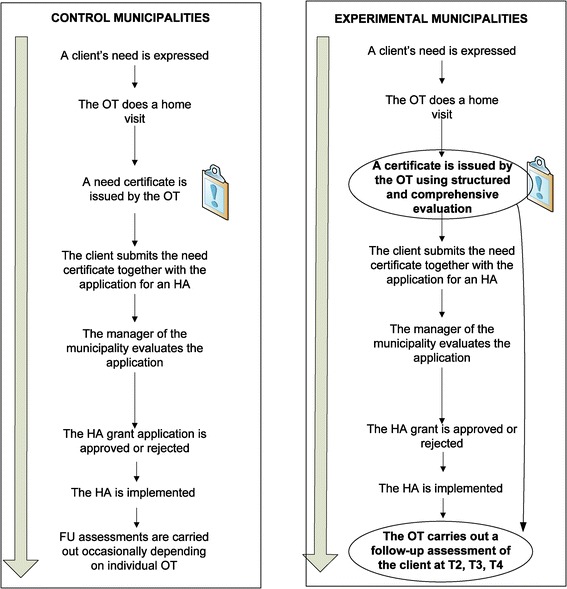
**Flow**-**chart of the HA process in the control and intervention municipalities.**

This quasi-experimental trial with a non-equivalent control group uses a before-after design [21]. The study applies a mixed-methods design [22] that combines quantitative and qualitative data. At the experimental sites, OTs apply an intervention that consists of a standardized research-based strategy for HA case management. That is, at the experimental sites, the OTs must change their HA practices, whereas at the control site, the OTs work according to their regular HA routines. The HA process at the experimental and control sites is described in Figure 1. Owing to the nature of the intervention, there is no blinding to study the condition assignment. This applies to the study participants, those administering the interventions, and the assessors.

At all the sites, identical data on outcomes are being collected at the same four time points: at T1 (before the HA) and then 3, 6, and 12 months after the HA (T2, T3, and T4, respectively). The data-collection methods are structured interviews, observation, and clinical assessments, as well as examining documents, such as grant proposals, certificates, grant decisions and invoices related to each client’s HA.

Quantitative and qualitative data from surveys, observations, interviews, and certificates will be collected and analyzed separately and combined with case studies, both cross-sectionally and longitudinally. Applying different mixed-methods research designs, data will be merged and combined in order to respond to the different research questions. For the qualitative research the RATS guidelines have been applied [23].

The study has been approved by the Ethical Review Board in Lund, Sweden (Dnr: 2012/566).

### The settings

Three municipalities in south Sweden will be included in this study: two experimental sites and one control site. We selected the municipalities based on the number of their inhabitants, their geographical dispersion, and their similar organizational structures for HA administration. Furthermore, because of the project’s complexity and duration and the effort required (particularly at the experimental sites), it is also necessary that staff members and their leaders (on several organizational levels) express a sincere interest in participating in the study and a readiness to change their practices [24,25].

### Participants

In the intervention and control municipalities, all the OTs who conduct HA needs assessment and certification as part of their ordinary practice are eligible for inclusion in the study. These OTs are the ones who identify potential clients for inclusion in the study.

All the sites have identical inclusion and exclusion criteria in relation to clients. All non-institutionalized persons over 20 years of age who apply for a HA grant via one of the OTs employed by the municipality are systematically considered eligible to participate in the study. Exclusion criteria constitute living in sheltered housing and/or an inability to communicate or follow instructions in Swedish. After a client has contacted an OT in relation to a HA errand, the OT asks the client whether he or she is willing to participate in the study. Participation in the study is completely voluntary and the participants receive oral and written information about the study and are giving their informed consent by signing a paper. Withdrawal or declined participation in the study does not affect further services.

#### Sample size calculation

The power calculation is based on data from a longitudinal study of the HA process [4,5]. Current power estimates are calculated based on primary outcomes: (1) dependence in daily activities, measured by means of the Activities of Daily Living (ADL) Staircase [26] revised version [27]; and (2) usability in the home, measured by the Usability in My Home (UIMH) instrument [28,29].

According to formal calculation, a sample size of 117 people in each municipality is required in order to detect the effect of the intervention with a statistical power equal to 80%. This size is based on an expected improvement of 0.5 in the ADL Staircase score and one of 1.5 on the UIMH score. This calculation is based on the assumption that an HA is effective in both experimental and control municipalities but that the effect is greater at an experimental site.

Given that we expect a drop-out rate of 30% over time in both groups owing to frailty, a total of 170 participants will be recruited at both experimental sites and the same number at the control site. Based on data from previous research, we anticipate that approximately 50% of those will use MDs outdoors or indoors [4,5], thus allowing meaningful sub-group analyses.

For the explorative segment targeting users’ perspectives, 20 clients and 10 cohabitants will be strategically selected for qualitative interviews. The selection will be based on data collection at T1, and the ambition is to make the sample as diverse as possible [30] in terms of sex, age, and MD use. Ideally, 10 in 20 clients should be living alone, whereas the remaining 10 should be cohabiting. The 10 significant others of cohabiting clients will also be invited for individual interviews.

### The intervention

The intervention is the application of a standardized research-based practice strategy for HA case management.

The development of the intervention was directed throughout by the Medical Research Council’s (MRC) guidelines [31], including dividing the process into the following stages: developing, piloting, evaluating, and implementing. The intervention consists of a new practice strategy comprising standardized procedures for the assessment and evaluation of both person- and housing-related aspects. Strategy development was guided by theories and models from medicine and health—namely, the International Classification of Functioning, Disability, and Health (ICF) [2], occupational therapy [32], environmental gerontology [33], and current Swedish legislation governing housing design, HAs [3,34], and MD provision [35]. Given the strong focus on evaluation, as well as the strong relation of the intervention to current policy and legislation in Sweden and to the MRC model, we also apply the theories of practice for guiding programme evaluation that Shadish, Cook, and Leviton [36] have outlined. In order to realize an evaluation that can capture both clinical and economic perspectives, Øvretveit’s [37] work on evaluation of health intervention is combined with health-economic theory and models about the effectiveness, cost, and cost-effectiveness of the intervention [38].

The intervention involves conducting structured assessments with standardized measurements. The variables selected are important as outcomes of HA or are included based on the assumption that they contribute to the structure and focus of the OT assessment. Some variables are included for participant-descriptive purposes. The intervention at experimental sites consists of the following structured components:

- An extensive training course for OTs covering the standardized research-based practice strategy. The 5-day course extends over 3 or 4 weeks. The research project team is responsible for the course and for training the OTs. The course covers how to conduct a structured assessment; how to use comprehensive and validated instruments in a structured way; and the background, purpose, construction, and basic psychometric properties of each study instrument, along with real-life situation assessments. The training also covers procedures for selecting participants and gaining informed consent, and it addresses ethical issues. The training course includes pilot assessments of the OTs before they begin data collection.
- OT assessments of physical environmental barriers and housing accessibility prior to HA certification.
- OT assessment of client outcomes in terms of ADL dependence, need for formal and informal help in the home, and MDs prior to HA certification.
- Client’s self-administered assessments of home usability, fear of falling, retrospective falls, participation and satisfaction, client goal fulfilment, and health-related quality of life prior to HA certification.
- Follow-up on client outcomes (i.e., according to OT assessments and self-reports) at home visits that take place 3, 6, and 12 months after HA implementation.

To strengthen data validity and reliability and to facilitate the incorporation of the intervention into ordinary practice, the following operations are planned:

- Project leaders will arrange regular seminars to increase compliance and adherence to the study protocol. The seminars aim to deepen OTs’ understanding of the intervention components and their competence in assessment and data analysis. One seminar in the early phase is organized as a focus-group interview, followed by additional focus-group meetings after 6 and 12 months.
- Individual contacts with OTs are undertaken via telephone or workplace visits for support and to clarify any questions that arise.

### The control municipalities

In the control municipalities, the OTs will not receive any specific training; they work according to their regular routines for HA case management. Thus, needs assessment will not necessarily employ instruments, follow-ups will be carried out occasionally (depending on local routines), and each OT will be free to register and manage client records according to the traditional structure used in the municipality. All the clients who meet the inclusion criteria and who have agreed to participate in the study will be contacted by a project administrator (i.e., a trained OT) who collects all data. Since the project administrator will do assessments in the control municipality similar to those the OT conducts at the experimental sites, the control group will receive more attention than usual, but not from a professional involved in the HA or other interventions. That is, the clients and their cohabitants receive the same amount of attention in both municipalities.

### Outcomes and measurements

Primary and secondary outcomes have been identified based on previous research findings, as outlined earlier, as well as based on current Swedish legislation concerning building design and HA.

Table 1 provides an overview of all the outcomes measures and other types of data collected.

**Table 1:** **Outcome measures and follow ups**

**Primary outcome**	**Measurements**	**Baseline**	**3 months**	**6 months**	**12 months**
		**(T1)**	**(T2)**	**(T3)**	**(T4)**
Usability of the home	Usability in My Home instrument [28,29].	X	X	X	X
ADL dependence and difficulty	ADL Staircase [26,41].	X	X	X	X
**Secondary outcome**					
Fear of falling	The Short Falls Efficacy Scale-International [42].	X	X	X	X
Fall incidence	Retrospective self-report on falls incidences during the last six months	X	X	X	X
Mobility devices	Self-reported use and need	X	X	X	X
Participation and satisfaction	Self-reported aspects of participation and satisfaction.	X	X	X	X
Health-related quality of life and quality adjusted life years (QUALYs)	EQ-5D-5L [47].	X	X	X	X
Cost	Item on costs for staff, material, travel, administration, etc.	X	X	X	X
Repeated HA	Self-reported question and register data from the municipality		X	X	X
Relocation	Self-reported question		X	X	X
Formal and informal care	Items on formal and informal care, who, and minutes per week, self-reported	X			X
**Descriptive variables**					
Age, gender, country of origin, educational level, employment, and civil status.	Self-reported questions	X			
Living conditions and housing standards		X	X	X	X
Cognitive status	Montreal Cognitive Assessment, MOCA [49,50].	X			
Functional limitations	Housing Enabler [48].	X	X	X	X
Environmental barriers	Housing Enabler [48].	X			

List of abbreviations: *ADL* Activities of Daily Living, *EQ-5D* European Quality of Life-5 Dimensions, *HA* Housing Adaptation.

#### Primary outcomes

- Usability denotes the effectiveness of, efficiency of, and client satisfaction with a specific environment or technical device. It focuses on the performance of tasks and activities and the related perception of satisfaction [39]. Usability is also central in current Swedish building legislation; thus, it is an important outcome of an HA. It is measured with a revised version of UIMH [28,29]. The client assesses his or her satisfaction with the housing design in activity performance. The instrument comprises 18 items that reflect 18 different personal, instrumental, leisure, and work-related activities.
- ADL dependence and difficulty is an operationalization of independence and is thus related to the legislative framework for HA and measured with the ADL Staircase [26,40]. It comprises nine items on feeding, transfer, using the toilet, dressing, bathing, cooking, transportation, shopping, and cleaning. For items on which the participant is rated as independent, he or she is also asked to state whether the corresponding activity is performed with or without difficulty [41].

#### Secondary outcomes

Client level

- Fear of falling is measured using the short form of the Falls Efficacy Scale-International [42], which consists of seven items (i.e., activities).
- Falls are targeted based on previous research [43] and measured using structured questions that concern frequency, fall rate, locations, injuries [44], and the frequency of near falls [45].
- Participation and satisfaction are assessed by means of study-specific questions based on previous research [46] and on the current goals of HA. This includes responding to 8 statements concerning the frequency of and client satisfaction with performance-related participation and togetherness-related participation.
- Health-related quality of life is measured by means of the EQ-5D-5 L [47]. This is a standardized instrument consisting of a descriptive system targeting mobility, self-care, usual activities, pain/discomfort, and anxiety/depression, in combination with the EQ visual analogue scale.
- Data on the type of MD and the frequency and location of its use are collected by means of the personal component of the Housing Enabler (HE) instrument [48], as well through questions used in previous research [43].
- Client goal fulfillment is investigated by means of open questions concerning specific goals and achievements with the HA before and after the intervention to the client and occupational therapist.

Societal level

- Health-economic data are important for societal planning purposes and in this study are collected in the following direct-cost categories: time spent by the OT with each client (both preparation time and direct contact); time other professionals spend with each client; time spent training the OT at the intervention site and other direct costs for training; time and other costs for travel; the cost of other consumables used for each client; the amount of the grant given to each client; and time for the assistance provided by formal and by informal (family) caregivers. Data is collected from an ad hoc resource-use questionnaire compiled by the OTs, by during the follow-up period retrieving grant applications, certificates, invoices and formal grant decisions, related to each case from the municipality office, and by using client-reported data on assistance received by informal caregivers. Data from the EQ-5D-5L instrument can be used for cost-effectiveness calculations, as well.
- Client relocation data are targeted by using a single dichotomous item question. This outcome is included because relocation to another ordinary or sheltered housing facility is sometimes an alternative to HA or a solution when HA proves insufficient.

#### Descriptive client data

Descriptive data concern both participants and housing. Here we include data on the prevalence of physical environmental barriers, functional limitations, formal and informal care, living conditions, housing standards, repeated HA, age, gender, country of origin, educational level, employment, and civil status.

At baseline, the HE instrument [48] is used to determine the physical environmental barriers and accessibility problems. HE is a valid and reliable instrument for assessing and analyzing accessibility problems in housing. The instrument is administered in three steps, utilizing a combination of interview and observation: the first step targets the individual by assessing the presence of functional limitations (12 items) and dependence on mobility devices (2 items). The second step assesses the presence or absences of physical environmental barriers in the home and in the immediate outdoor environment (161 items). Finally, based on the assessments in the first two steps, the magnitude of accessibility problems caused by a particular combination of functional limitations, dependence on MDs, and environmental barriers can be calculated using existing algorithms.

In addition, the Montreal Cognitive Assessment (MoCA) is used as a brief cognitive screening tool [49,50]. The MoCA covers a wide range of cognitive functions, such as short-term memory, executive functions, visuospatial abilities, language, attention, concentration, working memory, and temporal and spatial orientation [50].

The first baseline visit, conducted prior to the HA’s implementation, takes approximately 90 minutes. Each of the three visits after the HA (i.e., at 3, 6, and 12 months) takes approximately 45 minutes. This new practice strategy requires that the OT meets each client at least four times in his or her home (T1–T4). The assessment tools used to structure the data collection will differ among these measurement points (see Table 1).

#### Process data

In order to explore and evaluate the process and thus to understand changes in outcomes in the intervention municipalities, multiple methods are used to collect empirical data. The data collection focuses on feasibility, perceptions, and experiences of the intervention by targeting multiple perspectives—namely, those of clients, cohabitants, and OTs. Qualitative, semi-structured interviews with selected participants (i.e., clients and cohabitants) will be conducted before and after the HA in order to gain deeper knowledge about the participants’ expectations and experiences, as well as about the results of the HAs. The interviews will address the following three areas: (1) the intended goal of the HAs; (2) the clients’ and cohabitants’ roles in the decision, how well their needs and desires are met, and how they perceive the communication with various stakeholders; and (3) their long-term experiences and perceptions of the HAs in relation to home and health. The interviews will be audiotaped and conducted by the first author in the participants’ homes.

At the very end of the 5-day training course, the OTs complete an evaluation form focusing on their readiness to apply the new strategy in their daily practice. Facilitation in terms of reflection is an essential part of applying the intervention, and while the individual evaluations, seminars, and focus-group interviews are part of the intervention, the data collected on these occasions are used in the process evaluation, as well. After each assessment occasion, the OTs note whether they have followed the study protocol and in what ways they diverged from it, along with the reason for any non-adherence. Data collected on internal and external drop-outs will also be used to evaluate processes.

### Data analyses

Cross-sectional and longitudinal data analyses will be conducted depending on the research questions and the data, including statistical analyses, health-economic analyses, and qualitative analyses. Qualitative and quantitative data will be sequentially analyzed, and case studies will be developed.

#### Effects on the client level

Statistical analyses will be conducted on the group and sub-group levels, and common descriptive, inferential, and exploratory statistics will be applied. Depending on the characteristics of the variables, parametric and non-parametric statistics will be used, and both variable-based and person-based statistical analysis approaches [51] will be considered. All statistical analyses will be finally decided upon and described in subsequent papers, depending on the specific research questions they target.

Differences between the intervention and control groups in primary and secondary outcomes will be analyzed cross-sectionally at baseline and 3, 6, and 12 months later. Changes over time in outcomes will be calculated by means of a sign test for nominal data, Wilcoxon’s test for ordinal data, and a t-test for interval data. For correlations between single variables, Spearman’s rank correlation will be used, whereas canonical correlations will explore relationships among multivariate combinations of variables [52].

Because the study has a structure in which participants are nested within the municipalities, we will use hierarchical mixed-effects regression models and multi-level modeling approaches to evaluate differences between the intervention and control groups in changes over time in primary outcomes, as measured by the ADL Staircase and UIMH instruments, and in all secondary outcomes. Potential relations between different primary and secondary outcomes will be investigated by means of correlation analyses and Structural Equation Modeling Techniques (SEM). A mixed-linear-effects model will be used to examine the relation between client outcomes and client, as well as environmental characteristics, before and after the HA. The method is a development of regression and ANOVA, allowing a model of “within-subject dependence.” In order to generate knowledge of different groups’ patterns of changes, we will conduct a person-oriented analysis.

Missing data will be imputed following the guidelines for each instrument.

#### Effects on the societal level

Cost, cost-effectiveness, and cost-utility analyses will be performed from the perspective of the municipalities. The analysis of costs will consider the following direct-cost categories in both the experimental and the control groups.

In order to estimate more correctly the resources used in the process of HA, the assistance provided by informal caregivers will also be assessed and considered in the cost analysis. The value assigned to an hour of informal caregivers’ assistance will be equal to the hourly salary of an unskilled care assistant.

The cost-effectiveness analysis will compare the costs and outcomes of the two trial arms. As outcomes measures we will use the ADL dependency change over the trial period and the number of institutionalizations and housing relocations. Because both the costs and the effects of the interventions will occur over a period of one year, it will not be necessary to adjust for the time delay or to discount the measures.

Three incremental cost-effectiveness ratios (ICER) will be calculated by taking the difference in costs divided by the difference in the three outcome benefits between the two groups: one unit of ADL score gain, one institutionalization avoided, one relocation avoided. All available data on client and societal levels will be used to identify the variables included in the analysis in order to answer the hypotheses. The ICER will represent the additional costs of gaining, through the intervention, an additional unit of these outcomes measures.

In a later step, probabilistic sensitivity analyses (PSA) will also be performed on the three models using the software TreeAge Pro 2009. Results from the PSA will be presented as an acceptability curve, graphically illustrating the probability that the intervention is cost-effective over a range of willingness-to-pay values.

A cost-utility analysis (CUA) will estimate the ratio between the cost of an intervention and the benefit it yields in terms of quality-adjusted life years. The PSA of this model will follow a procedure similar to that of the cost-effectiveness analysis.

#### Process exploration and evaluation

To explore the processes behind changes in outcomes on a client and a societal level, quantitative and qualitative data will be analyzed over time.

The qualitative interviews of the clients and cohabitants are exploratory in character and will describe the process of applying for an HA, installing an HA, and using an HA in everyday life. Because the data reflect a process, the analysis will follow the principles of grounded theory according to Charmaz [53].

For each client, HA grant certificates and other documents related to the grant application such as grant proposals, grant decisions and invoices, will be analyzed in order to gain knowledge about the nature of the HA applied for, whether or not HA was granted, and other considerations among the OTs, the grant managers, and the clients and cohabitants.

The type of each HA and formal and informal help will be analyzed quantitatively and combined with survey data and data on drop-out, as well as with qualitative interview data. In addition, quantitative and qualitative data will be combined using mixed-methods analyses [22] in the context of case studies [54].

The process among the OTs will be analyzed by means of the baseline 30-minute paper in which the OTs describe in detail their current HA practices, the evaluation form focusing on their readiness to apply the new practice strategy, and the focus-group interviews. In addition, data about the quality of each OT’s application of the intervention in ordinary practice and on his or her study protocol adherence will be analyzed. For this purpose we will apply descriptive statistics, in combination with conventional and directed content analysis [55].

### Time frame

The project runs from 2013 to 2015.

## Discussion

The future evaluation of this non-randomized, quasi-experimental trial aims to determine the effects of applying a research-based strategy in relation to HA on the client and societal levels. One could argue that it would have been preferably to conduct a randomized control study (RCT) design. However, conducting a RCT in relation to HA is not possible in Sweden since those that are assessed as requiring HA have a legal right to receive it; they can therefore not be randomized to a non-intervention group. A RCT study in this area may however be possible in countries with other health care systems and legislations. For example, in the United States of America a prospective randomized controlled pilot trial design was used to evaluate the effect of a multi-component behavior and home repair intervention [56]. The control group then received the same amount of time as the intervention group did, spent on social attention and engagement instead of behavior and home repair intervention [56]. In the current study, the project administrator is spending the same amount of time with the participants in the control municipalities as the OTs is at the experimental sites. However, it is not possible to decline the participants in the control group the HA. Due to the Swedish context other designs than RCT have been used to evaluate the effect of HA by comparing groups receiving HA such as using people on the waiting list as a control group [8,57] or as in the current study by comparing ordinary practice to a structured research-based strategy.

The new practice strategy is based on extensive research conducted over the years, including both our own research [4–7,20,29,41,43] and that of other researchers [56,57]. The intervention in this study has been developed and tested through many years of research and in discussion with practitioners. This process has included methodological development and testing see, e.g., [29,58,59], research aimed at identifying the most important outcomes see, e.g., [6–8,18,46,57] and research targeting current HA case-management procedures in Swedish municipalities [20]. Since complex interventions comprise multiple components that are interrelated or interdependent they can be challenging to develop, document, evaluate, and report. However, based on knowledge on the wide variation of how the OTs structure their work on HA [20] studies like these are needed to develop evidence based practice.

The assessment instruments used have been developed and tested in various earlier projects. They have been psychometrically tested and are valid and reliable for use in research and practice in the context of the home, including considerations of HAs and MDs. Extensive assessment is part of the intervention, aiming not only at collecting data on important outcomes but also at focusing on the OTs’ pre-intervention assessment of relevant areas of the housing. This is the particular role of the HE [48]; an instrument that measures the magnitude of accessibility problems in housing. Physical environmental barriers are then assessed according to national standards for housing design. While these aspects are important, given that HAs are individually tailored interventions that extend beyond measures and standards, from a theoretical perspective norms and standards are not valid outcomes of HA [3]. However, using the HE as a valid and reliable checklist for the pre-HA walk-through assessment of the housing is extremely valuable and ensures that the OTs cover every area of the house in the walk-through.

One important aspect of health-care practice comprises the economic effects of various interventions. Health-economic evaluations call for data collected over a longer period, and the 12-month follow-up time frame applied in this study may be too short. Nevertheless, given that the health-economic data collected are more detailed and extensive than data gathered in previous Swedish studies on HA, this project will contribute to knowledge about the health-economic effects of HAs and MDs. Part of the intervention in this project is the data collectors’ education and training, which includes regular seminars and meetings for progress follow-up. Current research has demonstrated that in order to reach sufficient intervention fidelity, continuous data-collector monitoring is crucial, in particular in the case of complex interventions [60]. An intervention like this is time-consuming, both when it comes to the training of the occupational therapists and the time they spend doing the intervention, but might be efficient in the long term. This is important to take into account in the health-economic evaluation.

Once the study has been completed, the results will be used for further optimization of the practice strategy for HAs, in close collaboration with the data-collecting occupational therapists. Testing this new strategy in municipality practice responds to the need for effective health-promotion interventions that are based on research and provided in an efficient way.
